# Prescription patterns and effectiveness of medications for chronic obstructive pulmonary disease: A retrospective study of real-world settings

**DOI:** 10.1371/journal.pone.0304362

**Published:** 2024-06-10

**Authors:** Hye Jung Park, Jae Uk Lee, Soyoung Jeon, Hye Sun Lee, Bo Yeon Kim, Yu Jin Chae, Gui Ok Kim, Jung-Won Park, Jae-Hyun Lee

**Affiliations:** 1 Department of Internal Medicine, Gangnam Severance Hospital, Yonsei University College of Medicine, Seoul, Republic of Korea; 2 Biostatistics Collaboration Unit, Yonsei University College of Medicine, Seoul, Republic of Korea; 3 Healthcare Insurance Review & Assessment Service, Wonju, Republic of Korea; 4 Division of Allergy and Immunology, Department of Internal Medicine, Republic of Korea; 5 Institute of Allergy, Yonsei University College of Medicine, Seoul, Republic of Korea; Universiti Putra Malaysia, MALAYSIA

## Abstract

This study aimed to define real-world prescription patterns in Korea and compare the effectiveness of chronic obstructive pulmonary disease (COPD) medications. We used national claims data provided by the Health Insurance Review and Assessment Service in Korea and examined patients who were first diagnosed with COPD and started treatment between May 1, 2017, and April 30, 2018, with no change in drug regimen. Among 30,784 patients with COPD, long-acting β_2_ agonist (LABA) combined with long-acting muscarinic antagonist (LAMA) (32.7%), inhaled corticosteroid-LABA (ICS–LABA) (25.6%), LAMA (18.3%), ICS (5.8%), or LABA (4.6%) were prescribed as the first-choice inhalers. The use of LABA–LAMA (hazard ratio [HR], 0.248–0.584), LAMA (HR, 0.320–0.641), ICS–LABA (HR, 0.325–0.643), and xanthine (HR, 0.563–0.828) significantly reduced the total and severe exacerbation rates compared with no use of each medication. However, the use of ICS or LABA individually did not yield such effects. The continued use of LABA–LAMA, LAMA, and ICS–LABA showed a significant effect on exacerbation rate, whereas the long-term use of ICS, LABA, and xanthine did not. Moreover, some high doses of ICS–LABA did not show significant effects. This real-world study revealed that LAMA and/or LABA could be the first choice of therapy, as recommended by recent guidelines. However, ICS, xanthine, and high-dose ICS–LABA are still being prescribed frequently as first-line drugs in Korea.

## Introduction

Chronic obstructive pulmonary disease (COPD) is a chronic inflammatory condition affecting the airways, characterized by persistent and often progressive airflow obstruction. The main pathophysiology of COPD involves expiratory airflow limitations and air-trapping [1], leading to lung hyperinflation and dynamic lung hyperinflation, contributing to dyspnea and activity limitations, and thereby causing a decline in patients’ quality of life [1]. Furthermore, long-acting bronchodilators such as long-acting β2-agonists (LABA) and long-acting muscarinic antagonists (LAMA) are essential to relieve symptoms, improve quality of life, and prevent exacerbations of COPD [2]. LAMA and/or LABA remain the recommended first-choice drugs for patients with COPD [3, 4].

In addition to LAMA and LABA, other drugs are prescribed for patients with COPD. Inhaled corticosteroids (ICS) serve as anti-inflammatory agents and have been used to ameliorate inflammation in the airways. While ICS is not recommended as the first-choice drug in COPD [5], it can be adjunctively used with bronchodilators in selected patients, such as those at high risk of exacerbation, those with blood eosinophilia, or those who have COPD combined with asthma [6]. High-dose ICS can be an independent risk factor for the development of pneumonia and pulmonary tuberculosis; therefore, it is not generally recommended for COPD [7, 8]. Xanthine derivates, which represent an alternative COPD therapy, act as non-selective phosphodiesterase inhibitors and have additional benefits, such as changes in respiratory muscle function, improvement in arterial blood gas tension, and augmentation of the anti-inflammatory effects of corticosteroids [9]. Although the precise effects of xanthine remain controversial, it may still be prescribed to relieve symptoms.

Recent guidelines have recommend LAMA and/or LABA as first-choice drugs for COPD [4]. However, real-world data supporting this recommendation are lacking, and data on real prescription patterns in local clinics are insufficient. Therefore, this study aimed to define prescription patterns in patients with COPD in real-world clinical settings in Korea and compare the effectiveness of COPD medications. We obtained national claims data, which covers the entire Korean population and represents real-world medical data without any external intervention.

## Materials and methods

### Ethics

This study was conducted in accordance with the amended Declaration of Helsinki. Local institutional review boards or independent ethics committees approved the protocol. This study was approved by the Institutional Review Board of Severance Hospital (approval number: 4-2020-1355). The requirement for informed consent was waived due to the minimal risk and retrospective nature of the study.

### Data and participants

All medical institutions in South Korea claim medical expenses through the Korean Health Insurance Review and Assessment Service (HIRA), and this committee approves insurance reimbursement [10]. This insurance system covers almost all Korean citizens. The claims data for this national system provided by HIRA contains all medical visits and the drug regimens prescribed by medical institutions [11]. We analyzed the HIRA claim data recorded between May 1, 2016, and April 30, 2019. Data were accessed for research purposes from March 1, 2021, to December 31, 2021. Information that could identify individual participants were not accessed during or after data collection.

The definition of COPD has been previously described [12]. Briefly, we used diagnostic and prescribed medication codes according to HIRA data [13, 14]. Patients who met both the following criteria over 1 year were considered to have COPD:

1. ICD-10 codes for COPD or emphysema (J43.0x−J44.x), except for J43.0 as a primary or secondary (within the fourth position) diagnosis.
2. Administration of more than one of the following COPD medications at least twice per year:
  - LAMA
  - LABA
  - Fixed-dose ICS with LABA
  - Short-acting muscarinic antagonist
  - Short-acting β2 agonist
  - Short-acting muscarinic antagonist with short-acting β2 agonist
  - Phosphodiesterase-4 inhibitor
  - Systemic β agonist
  - Methylxanthine

We divided the time frame of this study into three periods: 1) a period to screen participants for COPD diagnosis (study period), 2) a period to exclude patients with COPD who had a history of COPD other than their first diagnosis (wash-out period), and 3) a period to evaluate the effects of the drugs used by the participants (assessment period). Patients who were first diagnosed with COPD and started treatment between May 1, 2017, and April 30, 2018 (study period), with no change in drug regimen, were selected. We excluded patients who used COPD medication between May 1, 2016, and April 30, 2017 (wash-out period) to exclude patients with a history of COPD. In addition, we excluded patients who changed their regimen between May 1, 2017, and April 30, 2019 (assessment period) to minimize the confounding effects of multiple drug use. During the assessment period, the clinical outcomes of COPD were assessed.

### Period of COPD medication use

We calculated the duration of COPD medication use by adding the number of days of COPD prescriptions during the study period. We classified COPD medication users according to the period of use as follows: < 3 months, 3–6 months, 6–9 months, and ≥ 9 months. In all subgroup analyses, we excluded subgroups with < 100 patients [10].

### Charlson’s comorbidity index

Charlson’s Comorbidity Index (CCI), which predicts disease prognosis, was calculated to adjust for underlying diseases, which may affect clinical outcomes [10]. Well-known underlying conditions, which affect mortality and prognosis, were assessed using diagnostic codes from the claims data between May 1, 2017, and April 30, 2018.

### Clinical outcomes

The primary clinical outcome was COPD exacerbation, defined as admission to the medical center (outpatient or inpatient) with COPD symptoms (according to the ICD-10 code of COPD [J43 and J44]) resulting in the administration of systemic corticosteroids for treatment. We assessed first and second occurrences of acute exacerbations and the exacerbation-free period for Cox regression analysis. Severe exacerbation was defined when exacerbation led to inpatient treatment.

### Statistical analysis

As in our previous similar work concerning asthma [10], we generated a time variable from the first date of COPD medication use to the date of the event (i.e., first or second acute exacerbation of COPD) and defined this as the exacerbation-free period. Considering this time variable, we employed Cox regression analysis to reveal the comparative effects of COPD medications. The control group was defined as non-users of specific COPD medication in all analyses (for example, we compared aclidinium users with non-aclidinium users, who might use other COPD medications). In addition, age, sex, and CCI were adjusted for in all Cox regression analyses. The data were analyzed using SAS Enterprise version 6.1 (SAS Institute Inc., Cary, NC,). A *P*-value < 0.05 was considered to indicate statistical significance.

## Results

### Study participants and baseline characteristics

**Fig 1:**
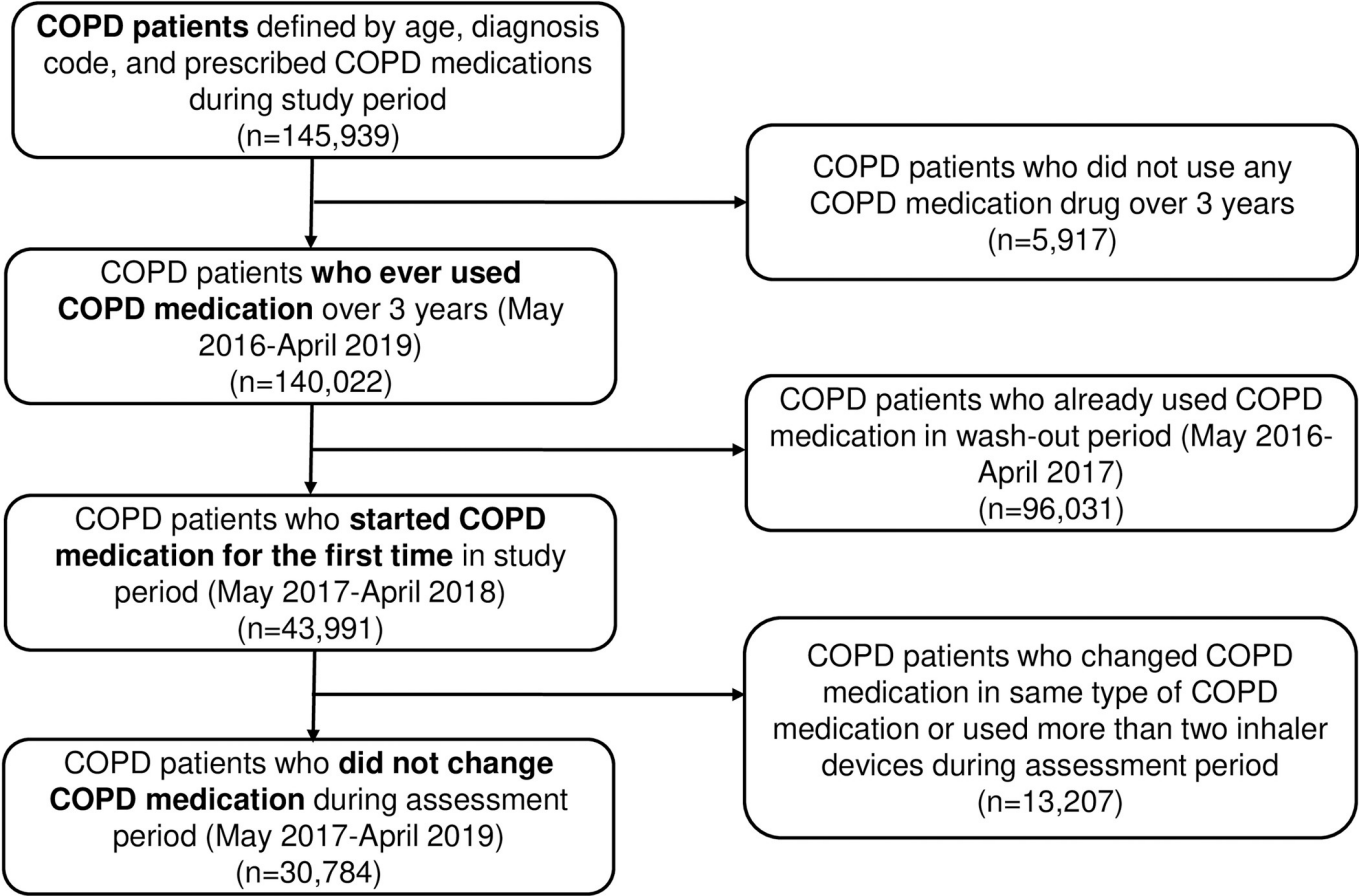
Study design and period.

During the study period, 145,939 patients with COPD were assessed. Of these, 140,022 used the study drugs (LAMA, LABA, ICS, ICS–LABA, LABA–LAMA and/or xanthine derivatives). After excluding patients who were already administered a study drug during the wash-out period, 43,991 treatment-naïve patients in the study period were diagnosed with COPD for the first time. We further excluded 13,207 patients who changed their regimen or used more than two inhaler devices during the assessment period. Finally, we analyzed the data of 30,784 patients with COPD to define the effects of COPD medication, to obtain a homogeneous sample and to minimize confounding factors (Fig 1).

### Baseline characteristics and outcomes of study participants

Of the 30,784 patients with COPD, LABA–LAMA (32.7%) was the most prescribed, followed by ICS–LABA (25.6%), LAMA (18.3%), ICS (5.8%), and LABA (4.6%). Xanthine derivatives were also prescribed frequently (55.9%), and 4,017 (13.0%) patients were prescribed only xanthine without inhalers. In total, 8,478 patients (27.5%) had COPD exacerbations, and 3,940 patients (12.8%) had severe exacerbations during the assessment period. Overall, 1,458 (4.7%) patients required admission to the emergency room. The total mortality was 4.8% (Table 1).

**Table 1:** Baseline characteristics and outcomes of study participants.

Characteristics		N (%)
Total		30,784 (100.0)
Sex	Male	21,736 (70.6)
	Female	9,048 (29.4)
Age	40–49	1,142 (3.7)
	50–59	4,024 (13.1)
	60–69	8,902 (28.9)
	70–79	10,924 (35.5)
	80–89	5,326 (17.3)
	90–99	460 (1.5)
	100-	6 (0.0)
CCI	1	6,237 (20.3)
	2	6,644 (21.6)
	Above 3	17,903 (58.2)
Medications		
Inhalers	LAMA	5,619 (18.3)
	LABA	1,407 (4.6)
	LABA-LAMA	10,074 (32.7)
	ICS	1,780 (5.8)
	ICS-LABA	7,887 (25.6)
Oral medications	Xanthine	17,214 (55.9)
Outcomes		N (%)
Total exacerbation		8,478 (27.5)
Severe exacerbation		3,940 (12.8)
ER admission for COPD exacerbation		1,458 (4.7)
Total Mortality		1,476 (4.8)

* CCI, Charlson’s comorbidity index; ER, emergency room

### Outcomes based on COPD medication

**Fig 2:**
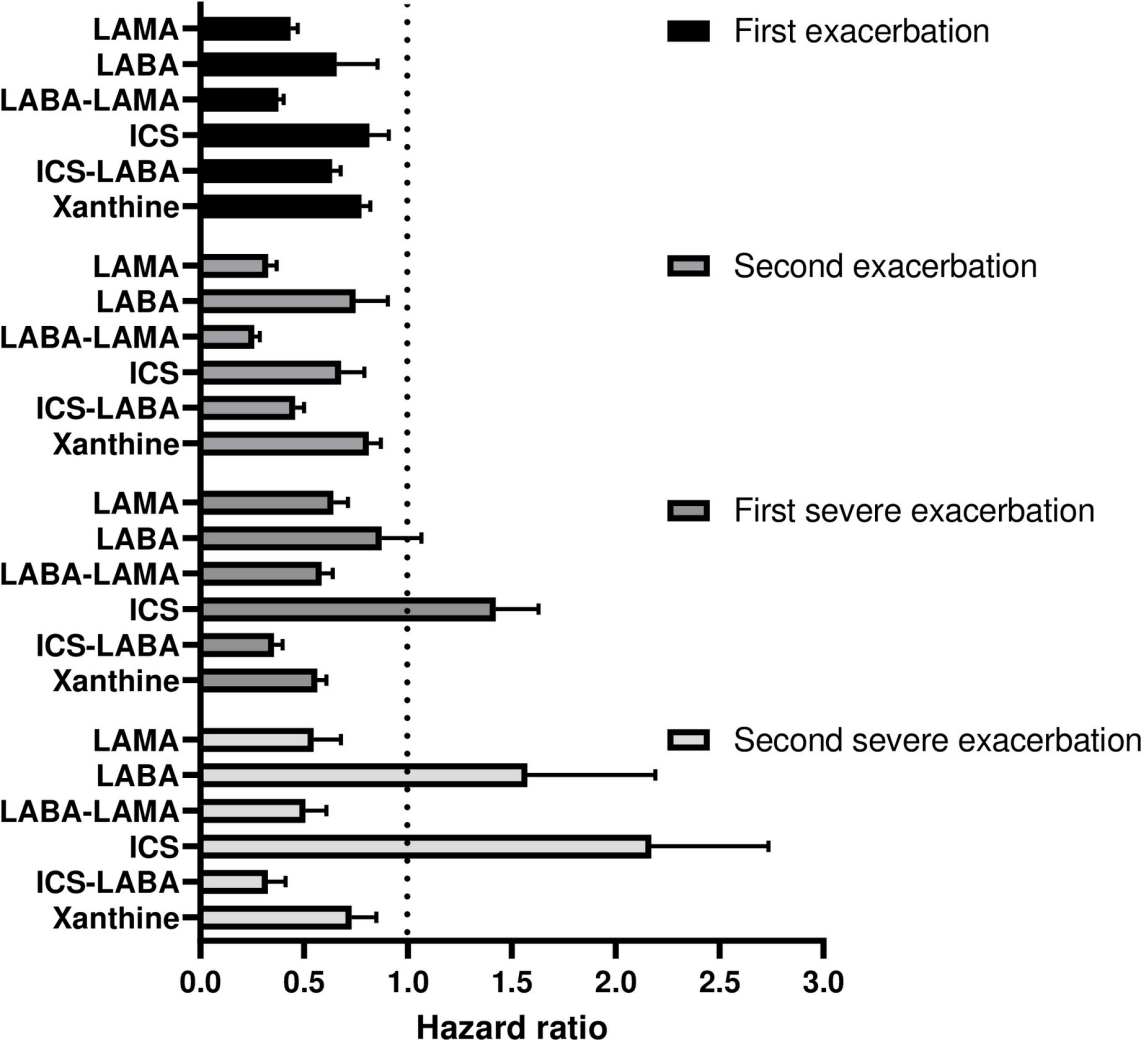
Hazard ratio for exacerbation according to COPD medication.

LABA–LAMA was the most effective drug combination to reduce the risk of first exacerbation (hazard ratio [HR], 0.376; 95% confidence interval [CI], 0.353–0.401; *P* < 0.001), followed by LAMA (HR, 0.434; 95% CI, 0.402–0.470; *P* < 0.001), ICS–LABA (HR, 0.634; 95% CI, 0.596–0.675; *P* < 0.001), LABA (HR, 0.657; 95% CI, 0.566–0.763; *P* < 0.001), xanthine (HR, 0.776; 95% CI, 0.736–0.818; *P* < 0.001), and ICS (HR, 0.814; 95% CI, 0.730–0.908; *P* < 0.001). All drugs significantly reduced the risk of second exacerbation (all *P* < 0.001). LABA–LAMA, LAMA, ICS–LABA, and xanthine significantly prevented the first and second severe exacerbations (all with *P* < 0.001). However, LABA and ICS did not demonstrate significant exacerbation prevention effects (Fig 2).

### Steady use of COPD medication

**Fig 3:**
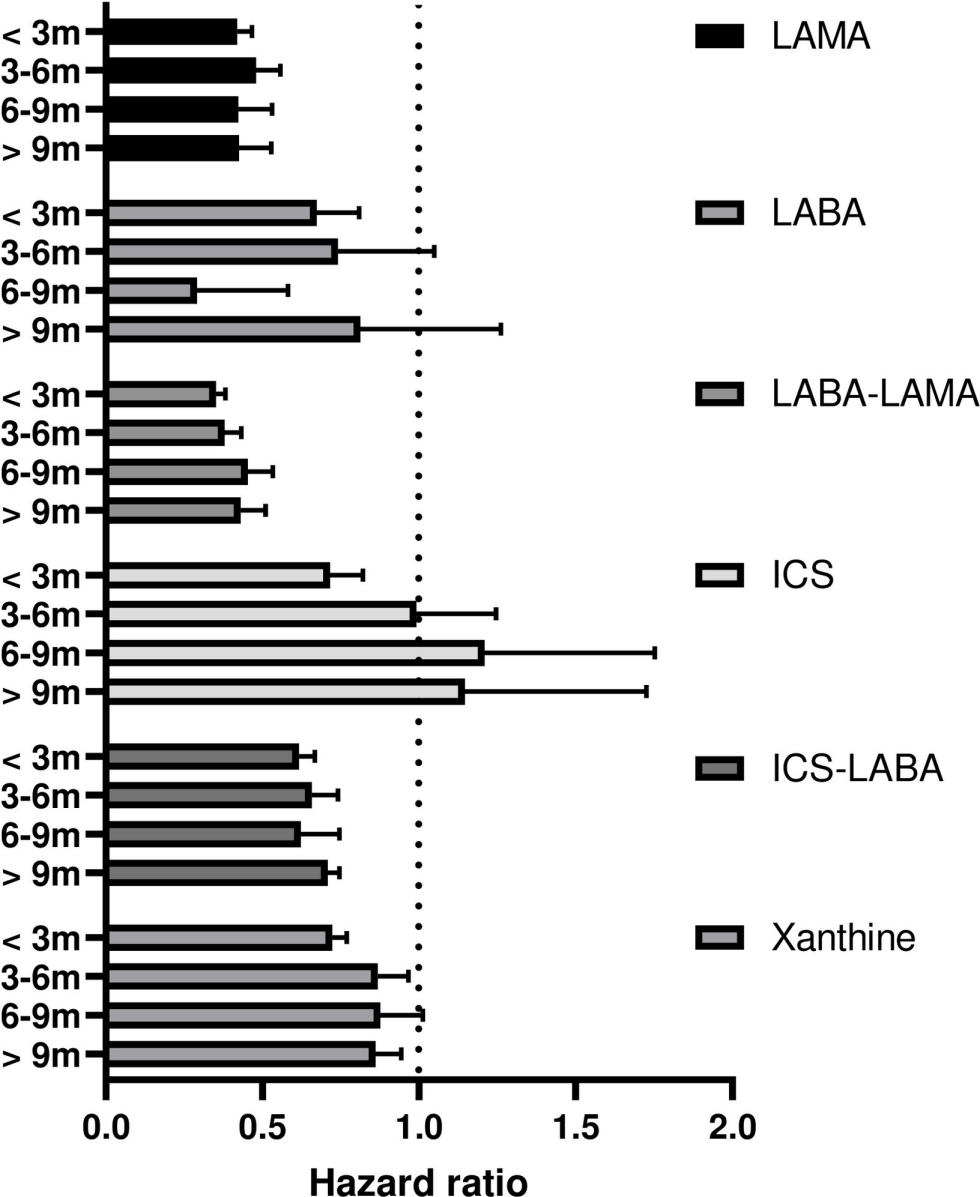
Hazard ratio for exacerbation according to period of medication use.

LABA–LAMA was the most effective drug combination for prevention of the first exacerbation, regardless of whether it was used for less than 3 months (HR, 0.351; 95% CI, 0.323–0.382; *P* < 0.001), 3–6 months (HR, 0.379; 95% CI, 0.332–0.433; *P* < 0.001), 6–9 months (HR, 0.453; 95% CI, 0.385–0.533; *P* < 0.001), or more than 9 months (HR, 0.430; 95% CI, 0.363–0.510; *P* < 0.001). LAMA (HR, 0.419–0.480; all with *P* < 0.001) and ICS–LABA (HR, 0.616–0.709; all with *P* < 0.001) also showed steady exacerbation prevention effects regardless of usage duration. The short-term use of LABA, ICS, and xanthine showed significant exacerbation prevention effects but not when they were used for more than 3 months (Fig 3).

### Active ingredient of COPD medication

Among LAMAs, aclidinium (HR for first exacerbation, 0.367; HR for second exacerbation, 0.251), tiotropium (HR for first exacerbation, 0.428; HR for second exacerbation, 0.335), and umeclidinium (HR for first exacerbation, 0.478; HR for second exacerbation, 0.330) significantly reduced acute exacerbations of COPD (all with *P* < 0.001). Among LAMAs, aclidinium was the most effective active ingredient for preventing COPD exacerbation. LABA–LAMA (HR for first exacerbation, 0.335–0.467; HR for second exacerbation, 0.214–0.349) significantly prevented acute exacerbations (all with *P* < 0.001). Among them, glycopyrronium-indacaterol was the most effective active ingredient (HR for first exacerbation, 0.335; HR for second exacerbation, 0.214). Among ICSs, ciclesonide (0.373–0.523) showed better effects than budesonide (HR, 0.749–0.887). As for xanthine derivates, theophylline (HR, 0.685–0.716) was the most effective drug to reduce the exacerbation rate (Table 2).

**Table 2:** Clinical outcomes according to the active ingredient of chronic obstructive pulmonary disease medication.

			Exacerbation			
			First event		Second event	
Type	Ingredient	N	HR (95% CI)	*P*-value	HR (95% CI)	*P*-value
LAMA	Aclidinium	452	0.367 (0.277–0.486)	<0.001	0.251 (0.162–0.389)	<0.001
	Tiotropium	3819	0.428 (0.389–0.470)	<0.001	0.335 (0.293–0.384)	<0.001
	Umeclidinium	1348	0.478 (0.412–0.554)	<0.001	0.330 (0.262–0.414)	<0.001
LABA	Indacaterol	342	0.184 (0.115–0.293)	<0.001	0.160 (0.082–0.314)	<0.001
LABA-LAMA	UME_VIL	262	0.467 (0.337–0.649)	<0.001	0.349 (0.217–0.562)	<0.001
	ACL_FOR	4655	0.358 (0.327–0.393)	<0.001	0.250 (0.218–0.287)	<0.001
	TIO_OLO	2459	0.447 (0.399–0.500)	<0.001	0.322 (0.274–0.380)	<0.001
	GLY_IND	2698	0.335 (0.297–0.378)	<0.001	0.214 (0.177–0.258)	<0.001
ICS	BUD	1476	0.887 (0.790–0.995)	0.041	0.749 (0.639–0.878)	<0.001
	CIC2	236	0.523 (0.366–0.749)	<0.001	0.373 (0.214–0.650)	<0.001
ICS-LABA	BEC_FOR	1135	0.589 (0.503–0.689)	<0.001	0.461 (0.370–0.575)	<0.001
	BUD_FOR2	1503	0.671 (0.590–0.762)	<0.001	0.515 (0.430–0.615)	<0.001
	BUD_FOR3	141	0.729 (0.494–1.075)	0.111	0.628 (0.375–1.053)	0.078
	FP_FOR2	196	0.663 (0.469–0.936)	0.020	0.355 (0.199–0.633)	<0.001
	FP_FOR3	139	1.032 (0.734–1.450)	0.857	0.773 (0.483–1.236)	0.282
	FP_SAL2	1213	0.708 (0.616–0.813)	<0.001	0.553 (0.456–0.671)	<0.001
	FP_SAL3	213	0.720 (0.522–0.993)	0.045	0.538 (0.340–0.850)	0.008
	FF_VIL1	2655	0.573 (0.515–0.636)	<0.001	0.364 (0.309–0.429)	<0.001
	FF_VIL2	610	0.627 (0.510–0.772)	<0.001	0.463 (0.342–0.626)	<0.001
Xanthine	Aminophylline	1955	0.799 (0.712–0.896)	<0.001	0.861 (0.740–1.004)	0.056
	Bamiphylline	435	0.715 (0.559–0.914)	0.007	0.758 (0.544–1.054)	0.100
	Doxofylline	12026	0.796 (0.751–0.843)	<0.001	0.825 (0.763–0.893)	<0.001
	Theophylline	2798	0.685 (0.617–0.761)	<0.001	0.716 (0.621–0.825)	<0.001

* Adjusted by age, sex, and CCI using Cox regression analysis

** HR, hazard ratio; CI, confidence interval; ICS, Inhaled corticosteroid; LABA, long-acting *β*_2_-agonist; LAMA, long-acting muscarine antagonist; BUD, budesonide 100 μg; CIC1, ciclesonide 80 μg, CIC2, ciclesonide 160 μg, FP, fluticasone propionate 100 μg; FP2, fluticasone propionate 250 μg; BUD1, budesonide 80 μg; BUD2, budesonide 160 μg; BUD3, budesonide 320 μg; BEC, beclomethasone; FOR, formoterol; SAL, salmeterol; FP3, fluticasone propionate 500 μg; FF1, fluticasone furoate 100 μg; FF2, fluticasone furoate 200 μg; VIL, vilanterol; UME, umeclidinium; ACL, aclidinium; TIO, Tiotropium; OLO, olodaterol; GLY, glycopyrronium; IND, indacaterol

For ICS–LABA, the ICS dose was the most important factor influencing the risk of exacerbation. Compared to low- and medium-dose ICS–LABA, high-dose ICS–LABA was less effective in preventing exacerbation. High-dose budesonide-formoterol (HR, 0.628–0.729) showed weaker effects than low-dose (HR, 0.461–0.589) and medium-dose (HR, 0.515–0.671) budesonide-formoterol. Medium-dose fluticasone-formoterol significantly reduced the exacerbation rate (HR, 0.355–0.663), whereas high-dose fluticasone-formoterol did not (HR for first exacerbation, 1.032, *P* = 0.857; HR for second exacerbation, 0.773, *P* = 0.282). Fluticasone-salmeterol and fluticasone-vilanterol showed similar exacerbation prevention effects, regardless of the ICS dose, whereas high-dose ICS–LABA showed slightly better effects than low-dose ICS–LABA (Table 2).

## Discussion

This study demonstrated the real-world prescription patterns and clinical effects of COPD medications without any external interventions in Korea. The Global Initiative for Chronic Obstructive Lung Disease (GOLD) guides the management of patients with COPD and the prescription of COPD medications. These guidelines are based on randomized clinical trials with strict and strong interventions; therefore, it is necessary to check whether they are proven suitable using real-world data [4]. Consistent with recent guidelines (the 2023 version), LAMA and/or LABA showed the best preventative effects on exacerbation in this study. ICS or xanthine monotherapy, as well as high-dose ICS–LABA, which are not recommended in the guidelines, showed weak effects in this study; however, they were still being prescribed for 5.8%, 23.3%, and 14.0% of patients, respectively. These findings indicate the need to educate clinicians to alter their prescription patterns to fit global guidelines.

LABA and/or LAMA was the most effective drug combination in this study. An outdated version of GOLD (2020 version) recommended LABA or LAMA as a first choice for B, C, and D patient risk groups. LABA–LAMA was only considered for patients in group D risk group if they were highly symptomatic [3]. However, multiple randomized clinical trials have demonstrated that LABA–LAMA is superior to LAMA and LABA monotherapy in patients with COPD [15, 16]. Moreover, the 2023 GOLD guidelines recommend LABA–LAMA in almost all COPD risk groups as the first-choice medication. This real-world study also supports this recent updated recommendation.

LABA–LAMA was the most frequently prescribed COPD medication in Korea (32.7%), while outdated GOLD guidelines only recommended LABA–LAMA in selected cases. This might be explained by the following reasons. First, unlike the outdated GOLD guidelines, the Korean COPD guidelines published in 2018 recommended LABA–LAMA as the first-choice treatment in most COPD risk groups [6], promoting the use of LABA–LAMA among clinicians in Korea. Second, patients with COPD only visit the hospital when they are highly symptomatic, given the lack of health literacy about COPD. Lastly, unusual Korean drug prices might affect the prescription pattern. Actually, the prices of LABA and LAMA monotherapy are quite similar to that of the LABA–LAMA combination in Korea. For example, the prices of umeclidinium alone, umeclidinium-vilanterol, and umeclidinium-vilanterol-fluticasone are 38,438 won (28.77$, calculated on March 1, 2024), 45,578 won (34.12$), and 45,602 won (34.13$), respectively (published at the homepage of the Ministry of Food and Drug Safety in Korea [http://www.mfds.go.kr]).

ICS monotherapy did not show a significant COPD exacerbation prevention effect in this study; however, some clinicians still prescribed it. ICS is an anti-inflammatory drug and can ameliorate airway inflammation [17]; however, it is known to increase the risk of pneumonia in patients with COPD [18]. Development of pneumonia can be a major risk factor for the COPD exacerbation and vice versa [19]; therefore, global guidelines do not recommend ICS alone as first-choice treatment for COPD. ICS monotherapy has rarely been prescribed for COPD by pulmonology specialists (0.2%) [20], but our study showed surprising proportions in the prescription rates of ICS as a first choice in Korean clinics (5.8%). Previously, Park et al. reported a high prescription rate (10.1%) of ICS monotherapy in primary clinics for COPD patients [21]. These findings indicate the need to educate clinicians, especially primary clinicians, not to prescribe ICS monotherapy in patients with COPD.

High-dose ICS–LABA showed weak clinical effects compared to other doses of ICS–LABA. High-dose ICS is not generally recommended for COPD. Only some studies have revealed that short-term high-dose ICS can have benefits in selected patients (those with increased eosinophilia and frequent exacerbations) [22]. Multiple studies have demonstrated that high-dose ICS can lead to pneumonia and tuberculosis in COPD, and well as to a poor prognosis [8, 23]. However, in this study, many clinicians prescribed high-dose ICS (41.5% as fluticasone-formoterol, 18.7% as fluticasone-vilanterol, 14.9% as fluticasone-salmeterol, and 8.6% as budesonide-formoterol) for patients with COPD. This enormous proportion of high-dose ICS–LABA prescriptions may be explained by the familiarity of this drug combination in patients with asthma. We should not prescribe high-dose ICS–LABA as a first choice in patients with COPD except in selected cases.

Xanthine has been frequently prescribed and has shown significant effects in preventing COPD exacerbations. Xanthine was the first choice in 55.9% of patients with COPD in Korea; however, an inhaler was added for the majority of these patients (76.7%). Xanthine monotherapy was chosen in 4,017 cases (23.3% of xanthine users). Xanthine possibly acts as a non-selective phosphodiesterase inhibitor, and it has a range of non-bronchodilator actions. However, the exact clinical effects and active duration of xanthine remain controversial [24]. Global guidelines do not mention xanthine monotherapy as first-choice treatment for COPD but rather state that xanthine should not be used due to increased side effect profiles. Some studies have showed xanthine can lead to a poor prognosis in COPD and increase the mortality [25]. However, many clinicians still prescribe xanthine monotherapy for COPD in Korea. A Taiwanese study also showed the vast majority of family medicine doctors (92.4%) and chest specialists (71.6%) also prescribed oral bronchodilators without inhalers [26]. These findings indicate that inhalers should be prescribed as first-choice treatment, especially LAMA and/or LABA, instead of oral xanthine.

This study demonstrated that the steady use of COPD medications leads to a sustained clinical effect. COPD is a chronic airway condition requiring continuous management and treatment. Global guidelines recommend that patients with COPD should use medication daily. Previous studies have shown that COPD medications have long-term efficacy and safety without any evidence of increased drug tolerance [27, 28]. This study also supports the long-term effects of COPD medication, mainly LABA–LAMA and LABA.

The distinct characteristics of Korean healthcare services compared to those of other countries should be considered. In Korea, almost all citizens (97%) are enrolled in the National Health Insurance (NHI) program. Except for cosmetic surgery or some unproven therapies, patients pay only 5%–30% of the total medical costs to clinics or hospitals. Clinics and hospitals then submit claims to the Health Insurance Review & Assessment (HIRA) to obtain reimbursement (70%–95% of the total cost) [29]. Since the medical costs that patients have to pay are relatively low, compared to other countries, patients easily present to the hospital even with mild symptoms. In addition, drug cost is not an important factor in choosing monotherapy or combination therapy as drug prices do not vary greatly.

This study has several strengths. First, we strictly controlled for extrinsic factors and carefully selected new drug users, which may have possibly improved the validity of this study. Second, we used HIRA data, which covers the majority of Korean citizens. Lastly, we used Cox regression analysis while considering the exacerbation-free period. However, this study also has several limitations. First, we could not divide the data according to the type of inhaler devices. Second, we excluded some patients with COPD who changed prescriptions or used two or more medications; however, if we had included these, hundreds of groups would have been generated. We also excluded some unpopular COPD medications to minimize unreliable data (e.g., < 100 prescriptions). This can affect the generalizability of study findings in real-world settings. Third, COPD is a clinical disease confirmed by lung function test and clinical features; however, we defined COPD using diagnostic codes and medical history. This definition might lead to bias; however, this is the most frequently recommended and used method when HIRA data are used [12, 14, 30]. Fourth, we could not adjust for important clinical data, which can influence the severity and prognosis of COPD, such as smoking, COPD group, lung function, symptoms, and environmental factors. To overcome this limitation, we excluded COPD patients with a history of COPD exacerbation to exclude severe patients, to make the sample more homogenous and to prevent skewed outcomes. Lastly, there are inherent limitations associated with the use of prescription data: 1) the actual consumption of medications cannot be confirmed; 2) prescription patterns can be influenced by various factors such as physician and patient preferences, healthcare policies, and pharmaceutical marketing strategies; and 3) temporal relationships between treatments and outcomes might be inaccurate.

## Conclusion

This real-world study, which covered almost all patients with COPD in Korea, revealed that LAMA and/or LABA is the most effective treatment. ICS or xanthine monotherapy and high-dose ICS–LABA showed weak effects in this study. Therefore, LAMA and/or LABA should be the first-choice inhaler, as recommended in recent guidelines. ICS or xanthine monotherapy and high-dose ICS–LABA are still prescribed frequently as first-choice drugs in Korea; however, we need to reduce the prescription rate of these medications. We recommend changes in the prescription pattern of these drugs in accordance with recent guidelines. Further studies integrating prescription data with other sources such as electronic health records, patient-reported outcomes, and observational studies are warranted to strengthen this conclusion and to provide a more comprehensive understanding of treatment effectiveness in real-world settings.

## Abbreviations list

CCI: Charlson’s comorbidity index
CI: confidence interval
COPD: chronic obstructive pulmonary disease
GOLD: Global Initiative for Chronic Obstructive Lung Disease
HIRA: Health Insurance Review and Assessment Service
HR: hazard ratio
ICS: Inhaled corticosteroid
LABA: long-acting β_2_ agonist
LAMA: long-acting muscarine antagonist
OR: odds ratio
